# The Role of Exenatide, a Glucagon-Like Peptide-1 (GLP-1) Receptor Agonist, in Idiopathic Intracranial Hypertension and Polycystic Ovary Syndrome: A Case Report

**DOI:** 10.7759/cureus.93846

**Published:** 2025-10-04

**Authors:** Despina Nicolaou, Nicolas Nicolaou, Lisa M Douglas, Savvas Christou

**Affiliations:** 1 Obstetrics and Gynecology, Addenbrooke's Hospital, Cambridge University Hospitals NHS Foundation Trust, Cambridge, GBR; 2 Obstetrics and Gynecology, Peterborough City Hospital, Peterborough, GBR; 3 General Surgery, Addenbrooke's Hospital, Cambridge University Hospitals NHS Foundation Trust, Cambridge, GBR; 4 General Practice, Addenbrooke's Hospital, Cambridge University Hospitals NHS Foundation Trust, Cambridge, GBR; 5 Ophthalmology, St. George Hospital, Paphos, CYP

**Keywords:** exenatide, glp-1 receptor agonists, hyperandrogenism, idiopathic intracranial hypertension (iih), polycystic ovary syndrome (pcos), reproductive outcomes, weigthloss

## Abstract

Idiopathic intracranial hypertension (IIH) and polycystic ovary syndrome (PCOS) are strongly associated with obesity in women of reproductive age. Glucagon-like peptide-1 receptor agonists (GLP-1 RAs) have been extensively reported to facilitate weight loss through central satiety. Emerging evidence demonstrates additional benefits of GLP-1 RAs. Exenatide exerts direct effects on choroid plexus receptors, reducing cerebrospinal fluid (CSF) secretion and intracranial pressure (ICP), and on the ovary, enhancing follicular maturation, restoring ovulation, and improving fertility outcomes in PCOS. A 29-year-old woman with PCOS was referred from gynecology for assessment of intermittent visual blurring and chronic headaches. Ophthalmic examination revealed normal monocular visual acuity of 6/5 in each eye but reduced binocular vision of 6/12, associated with double vision. She also reported a weight gain of 15 kg over eight months. Fundoscopy was unremarkable, with no optic disc swelling. IIH without papilledema (IIHWOP) was diagnosed based on magnetic resonance imaging showing an enlarged pituitary fossa, partial empty sella, and optic nerve sheath dilatation, with lumbar puncture confirming a raised opening pressure of 280 mmH₂O and normal CSF. Initial treatment with acetazolamide was discontinued due to intolerance. Exenatide was subsequently initiated, leading to early improvement in headache frequency and diplopia, independent of weight loss. Menstrual cycles, hormonal profile, and insulin resistance also improved beyond the effect of metformin alone. This case highlights the potential of exenatide to provide dual therapeutic benefits in IIH and PCOS, acting through direct receptor-mediated pathways to reduce ICP, improve headaches, and restore ovulatory cycles, and indirectly through the reduction of central obesity. Further studies are warranted to establish its role in the combined management of these conditions.

## Introduction

Idiopathic intracranial hypertension (IIH) is a rare neurological disorder characterized by raised intracranial pressure (ICP) without a mass lesion or secondary cause [1]. It commonly presents with headache and optic disc swelling, though it may occur without papilledema (IIHWOP), and requires urgent treatment to prevent progressive optic nerve damage and irreversible visual loss [1,2,3]. Between 15.5% and 57% of patients with IIH also have polycystic ovary syndrome (PCOS) [1]. Both conditions are strongly associated with obesity and share overlapping hormonal features, most often in women of reproductive age. PCOS affects 4-10% of women in this group, while the incidence of IIH has risen from 2 to 9.3 per 100,000 over the past decade, reflecting the impact of increasing obesity [1]. Weight loss remains the cornerstone of management in both conditions, and glucagon-like peptide-1 receptor agonists (GLP-1 RAs) have been shown to achieve this through satiety mechanisms [4-7]. However, more immediate effects are often required to lower ICP. Acetazolamide, the main pharmacological treatment, is poorly tolerated in nearly half of patients [3]. Exenatide is a promising dual-purpose agent that has demonstrated both direct and indirect effects. Direct actions on GLP-1 receptors in the choroid plexus reduce cerebrospinal fluid (CSF) secretion and ICP, improving headache burden, while actions on the ovaries enhance follicular maturation, restore ovulation, and improve fertility [4,8,9]. In addition, exenatide has been shown to improve the efficacy of standard therapies such as metformin and sodium-glucose cotransporter-2 inhibitors, providing further metabolic and reproductive benefits [6,10,11,12 ]. We present the case of a woman with PCOS who developed IIHWOP following rapid weight gain, with symptoms of persistent headaches and visual blurring. Treatment with exenatide led to improvement of headaches within two days, a favorable hormonal profile, and restored menstrual cycles. This review highlights the potential of exenatide to provide dual benefits in IIH and PCOS. By addressing both direct receptor-mediated effects and indirect contributors such as insulin resistance and hormonal imbalance, exenatide may improve ICP regulation and reproductive outcomes.

## Case presentation

A 29-year-old woman presented with a six-month history of daily bilateral headaches refractory to medication. She also reported intermittent visual blurring, diplopia, and photopsia. Over the preceding eight months, she had experienced stress-related weight gain of 15 kg, resulting in a BMI of 31.2 kg/m², along with secondary amenorrhea for two months. She was diagnosed with PCOS with associated hirsutism, acne, hyperlipidemia, and insulin resistance. A combined oral contraceptive pill (COCP) was commenced, which led to mild improvement in PCOS symptoms; however, her headaches worsened. She was advised lifestyle modification for weight loss, but no pharmacological treatment was initiated.

**Figure 1:**
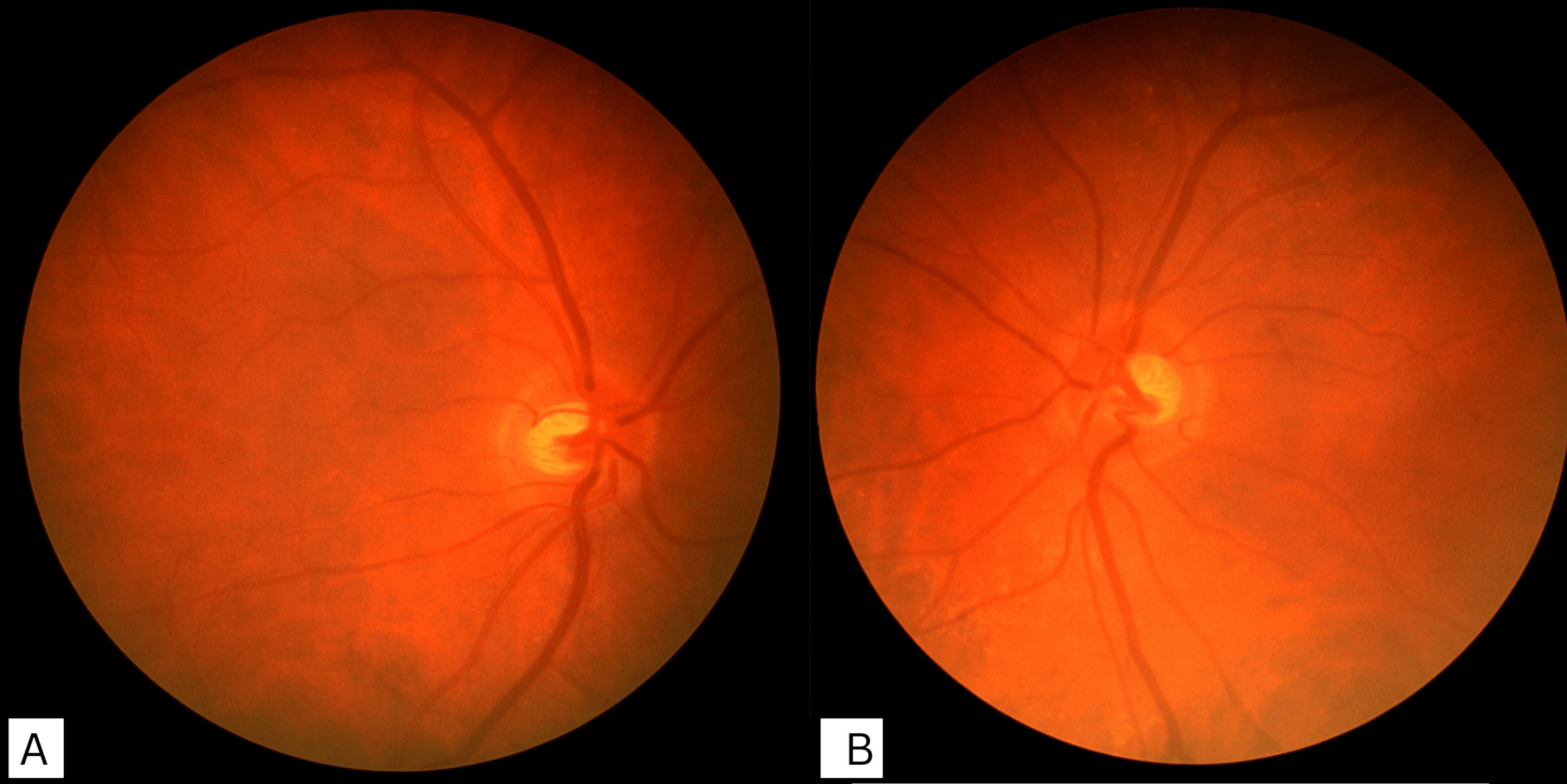
Fundus photographs of the optic discs. (A) Right eye and (B) left eye showing no optic disc swelling. Absence of papilledema, typically expected in idiopathic intracranial hypertension, is consistent with idiopathic intracranial hypertension without papilledema (IIHWOP).

**Figure 2:**
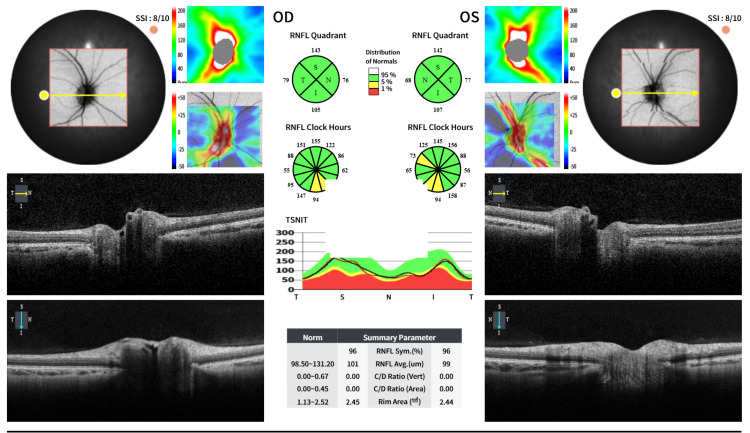
Optical coherence tomography (OCT) of the optic nerves in the right (OD) and left (OS) eyes. Optical coherence tomography (OCT) demonstrates normal peripapillary retinal nerve fiber layer (RNFL) thickness in both eyes. The optic nerves show preserved RNFL symmetry, with no evidence of thinning or swelling, consistent with the absence of papilledema.

She was referred from gynecology for ophthalmic assessment due to visual symptoms. Monocular visual acuity was 6/5 in each eye, but binocular acuity was reduced to 6/12. Extraocular movements were full, although convergence was moderately impaired. Color vision was normal on Ishihara testing, and there was no relative afferent pupillary defect. Fundoscopy was unremarkable with no papilledema, and automated perimetry demonstrated normal blind spot size. Optical coherence tomography (OCT) showed normal peripapillary retinal nerve fiber layer (RNFL) thickness (right: 96 µm; left: 95 µm). Neurological examination was otherwise normal (Figures 1-2). 

**Figure 3:**
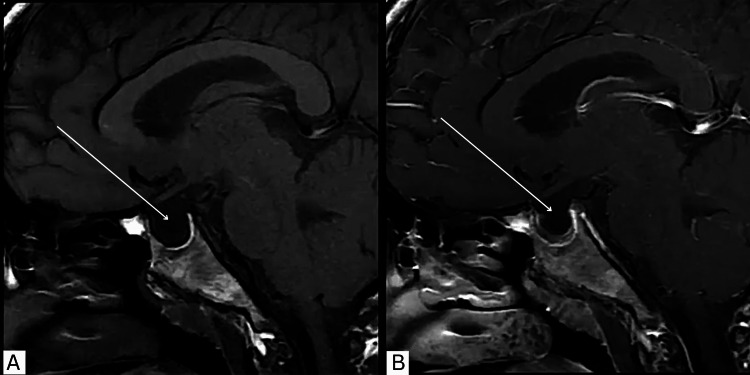
Partially empty sella on sagittal T1 magnetic resonance imaging (MRI). A sagittal T1-weighted (A) pre- and (B) post-contrast MRI showing a partially empty sella filled with cerebrospinal fluid (CSF) with a thin rim of pituitary tissue. An empty sella occurs when CSF herniates into the sella turcica, compressing the pituitary gland, creating the appearance of a partially or completely *empty* sella on MRI.

Vital signs were unremarkable (blood pressure 110/79 mmHg, heart rate 89 bpm) and hemoglobin was 12 g/dL. Magnetic resonance imaging (MRI) of the brain with MR venography was performed prior to lumbar puncture to exclude cerebral venous sinus thrombosis and space-occupying lesions. Imaging revealed an enlarged pituitary fossa with partial empty sella and optic nerve sheath dilatation, consistent with raised intracranial pressure (Figure 3). 

Lumbar puncture demonstrated an opening pressure of 280 mmH₂O with normal CSF composition, excluding infection and other secondary causes. Headache and visual symptoms improved significantly following the tap. Endocrine evaluation confirmed elevated total testosterone (hyperandrogenism) and an increased LH/FSH ratio, together with insulin resistance, consistent with PCOS. Overall, the clinical, ophthalmic, radiological, and CSF findings supported a diagnosis of IIHWOP associated with obesity . 

Management focused on weight reduction and intracranial pressure control. Acetazolamide 500 mg twice daily was initiated but was not tolerated and subsequently discontinued. Topiramate had only mild effect on headaches. Exenatide, a GLP-1 RA was then commenced at 5 µg subcutaneously twice daily and later increased to 10 µg twice daily. Initial nausea was reported but resolved with continued therapy. The patient noted improvement in headache symptoms within several days of starting exenatide. Metformin was subsequently added, with exenatide enhancing its effects on insulin resistance and regulation of hormonal imbalance in PCOS. Following a weight loss of 7.5 kg over four months, her headaches and diplopia-related visual blurring resolved completely.

## Discussion

IIH commonly presents with intractable headaches, nausea, pulsatile tinnitus, and progressive visual loss, along with bilateral optic disc swelling [1,2]. Our patient presented without papilledema or visual loss. IIHWOP, reported in 5.7% of patients with IIH, is now believed to be more common and should be suspected in obese women with chronic daily headaches refractory to conventional therapy [2,3]. In one headache clinic, 15% of patients were found to have raised ICP (>250 mm CSF) in the absence of papilledema [3]. IIHWOP has since been incorporated into the International Headache Society classification criteria [3]. Unlike IIH, IIHWOP usually spares vision. Diagnosis is based on raised CSF opening pressure with normal composition, absence of a secondary cause on imaging, and supportive radiological findings such as an empty sella or optic nerve sheath dilatation [2,3]. The absence of papilledema may be explained by structural variations in the optic nerve sheath and is no longer considered an exclusion criterion for IIH according to Friedman et al. [2,3]. 

The mean CSF opening pressure in IIHWOP (309 mmH₂O) is lower than in classic IIH with papilledema (373 mmH₂O; *P* = 0.031) [3]. In this case, the ICP was 280 mmH₂O, lower than typical IIH values but still above normal. Excessive weight gain over a short period is a recognized risk factor for IIH, as has also been reported in several pregnancy cases [1,4]. Other clinical features of IIHWOP often include photopsia, migraine-like auras, and intermittent visual blurring. Preserved monocular vision but reduced binocular vision, as in our patient, was due to diplopia caused by raised ICP compressing the abducent nerve and impairing convergence [2,3].

Acetazolamide, a carbonic anhydrase inhibitor, is the most commonly prescribed first-line therapy, providing a short-term reduction in ICP [3]. However, our patient declined treatment due to poor tolerance. Notably, in a randomized controlled trial (RCT), nearly half of patients (48%) discontinued acetazolamide within three months owing to adverse effects such as dysgeusia, paresthesia, fatigue, and nausea [4]. These findings emphasize the need for safer, more effective therapies to lower ICP.

Weight loss remains the cornerstone of management in IIH and a first-line intervention in PCOS [1,5]. A reduction of as little as 10% in body weight can lead to sustained improvement in IIH-related symptoms such as headache, visual disturbances, and papilledema [5]. GLP-1 RAs promote weight loss by enhancing glucose-dependent insulin secretion, suppressing glucagon release, and reducing appetite. Semaglutide is well known to achieve significant weight loss, beneficial in both PCOS and IIH, with the key advantage of once-weekly subcutaneous injection compared with the twice-daily dosing of exenatide [5,6]. However, its direct effects on CSF production remain largely unexplored. Exenatide has been more extensively studied, with evidence supporting its role in both conditions, making it a promising agent [4,5].

Beyond its metabolic actions, exenatide has been observed to reduce sodium reabsorption and promote diuresis via the renal proximal tubule, thereby lowering fluid retention [4]. In addition to this peripheral mechanism, preclinical studies have demonstrated central effects on CSF secretion. In vivo rodent models identified GLP-1 receptors in the choroid plexus, where activation with the GLP-1R agonist exendin-4 lowered ICP in hydrocephalic female rats. Further evidence for the functional presence of these receptors was provided by the GLP-1R antagonist exendin 9-39, which increased CSF secretion [4,7].

These findings were translated into a phase 2 RCT, where 15 women with IIH received subcutaneous exenatide or placebo, with ICP continuously monitored using telemetric intracranial pressure catheters. Exenatide significantly reduced ICP at 2.5 hours (−5.7 ± 2.9 cmCSF, P = 0.048) and 24 hours (−6.4 ± 2.9 cmCSF, P = 0.030), with a sustained trend at 12 weeks (−5.6 ± 3.0 cmCSF, P = 0.058), corresponding to circulating exenatide levels [4]. The rapid ICP reduction observed within hours is unlikely to be explained by weight loss. Instead, binding of exenatide to GLP-1 receptors in the choroid plexus, reducing Na⁺/K⁺-ATPase activity and decreasing CSF secretion, provides a more plausible mechanism [4,5,7]. Molecular evidence of GLP-1 receptor expression in the choroid plexus using RNAscope and Western blot was confirmed in a more recent rodent study. Exendin-4 reduced ICP again in hydrocephalic male rats, partly through Na⁺/K⁺-ATPase inhibition; however, the use of male models is a limitation, as IIH predominantly affects women [8].

More importantly, exenatide reduced ICP and significantly decreased monthly headache days (−7.7 vs −1.5 with placebo), and our patient similarly experienced early headache improvement. To date, no other off-label treatment for IIH has demonstrated such a rapid reduction in ICP within 24 hours, an important clinical advantage given the risk of sudden visual loss and neurological deterioration [4]. Further studies are needed to confirm the rapid ICP reduction observed with exenatide and to evaluate the efficacy of other GLP-1 RAs with longer dosing intervals.

In terms of safety, the RCT did not identify any concerns with exenatide [4]. A subsequent study assessed potential cognitive effects while lowering ICP, randomizing patients to exenatide (n = 7) or placebo (n = 8), with cognitive function evaluated using the NIH Toolbox Cognitive Battery at baseline and after 12 weeks. Exenatide reduced ICP without adversely affecting cognition, supporting its potential as a safe and effective therapy [9].

While several GLP-1 RAs have been used in IIH management, their role has largely been limited to complementary weight-loss therapy, with evidence primarily derived from indirect or observational studies. Exenatide is the only agent that has been evaluated in an RCT, directly demonstrating a rapid reduction in ICP through telemetric monitoring, alongside a marked reduction in headache days [4]. In contrast, data for liraglutide and semaglutide come from real-world or pilot studies, showing improvements in papilledema over 3 to 24 months, but outcomes relied on electronic health records without invasive ICP measurements, and the effects on headache and visual symptoms were less robust than with exenatide [5,10].

PCOS was diagnosed in our patient using the Rotterdam criteria, requiring two of oligo/amenorrhea, hyperandrogenism, or polycystic ovarian morphology on ultrasound [1,11]. Between 15.5% and 57% of patients with IIH also have PCOS, reflecting a close pathophysiological relationship with obesity [1]. Targeting abdominal obesity is therefore central to the management of both conditions. The main pharmacological therapies for PCOS are COCPs, which regulate menstrual cycles, and metformin, which improves insulin sensitivity. SGLT2 inhibitors may also improve metabolic outcomes in women with PCOS [13]. Emerging evidence suggests that these effects can be further enhanced with GLP-1 RAs [6,11-13].

GLP-1 RAs act on receptors in the hypothalamic arcuate nucleus, stimulating proopiomelanocortin (POMC) neurons that suppress appetite, delay gastric emptying, and reduce caloric intake. These mechanisms collectively promote sustained weight loss, reduce central obesity, and lower inflammatory mediators associated with PCOS [1,6,11]. Beyond their well-recognised effects on weight reduction, GLP-1 RAs also provide broader reproductive and metabolic benefits in women with PCOS.

PCOS is the leading cause of anovulatory infertility, driven by hormonal disturbances that impair follicle development. Elevated androgen levels inhibit dominant follicle growth, while excess adipose tissue promotes aromatization of androgens to estrogens. This process suppresses gonadotropin-releasing hormone (GnRH), luteinizing hormone (LH), and follicle-stimulating hormone (FSH) release via negative feedback, thereby reducing ovarian stimulation [1,6,11]. Insulin resistance further contributes to reproductive dysfunction, as compensatory hyperinsulinemia stimulates ovarian theca cells to produce excessive androgens while simultaneously suppressing hepatic synthesis of sex hormone-binding globulin (SHBG). The resulting increase in free circulating androgens sustains hyperandrogenism, disrupts the hypothalamic-pituitary-ovarian axis, and perpetuates anovulation and infertility [1,6,11,12].

GLP-1 receptors have also been identified in ovarian tissue, and animal studies suggest that GLP-1 RA may play a direct role in regulating follicular development and ovulation [12]. Clinical evidence supports these findings, with data from 27 RCTs in women with PCOS showing that GLP-1 RAs restore menstrual cyclicity, improve ovulation, increase pregnancy rates, and alleviate both metabolic and hormonal disturbances [12,13]. 

Exenatide, originally developed for type 2 diabetes and obesity, has the strongest body of evidence among GLP-1 RAs in PCOS, demonstrating reproductive and metabolic benefits [6,13]. By lowering testosterone while increasing FSH and SHBG levels, exenatide helps restore menstrual cycles and improve fertility potential. A systematic review and meta-analysis of nine RCTs (*n* = 1,059 women) found that exenatide was more effective than metformin in reducing total testosterone (TT) (SMD −0.43, 95% confidence interval (CI) −0.84 to −0.03, *P* = 0.04), improving FSH and SHBG (MD 5, 95% CI 3.82-6.18, *P* < 0.001), and increasing pregnancy rates (RR 1.85, 95% CI 1.19-2.86, *P* = 0.006), thereby enhancing reproductive outcomes [11]. In our patient, exenatide was used in combination with metformin. The same meta-analysis found that this combination provided greater improvements in SHBG (MD 10.38, 95% CI 6.7-14.06, *P* < 0.001), insulin sensitivity, free androgen index (FAI) (MD −3.34, 95% CI −4.84 to −1.83, *P* < 0.001), and body weight compared with metformin alone [11]. 

Sridharan et al. confirmed these findings, showing that the combination of GLP-1 RAs and metformin significantly reduced TT, FAI, androstenedione, and LH, while increasing SHBG and estradiol levels [13]. Consistent with these findings, Papaetis et al. reviewed several GLP-1 RAs, including liraglutide, exenatide, dulaglutide, and semaglutide. They too reported a reduction in free testosterone and FAI, an increase in SHBG, and improvement in menstrual frequency and ovulation rates, most notably with liraglutide and exenatide [6]

Exenatide significantly improves insulin sensitivity and reduces hyperinsulinaemia, thereby targeting one of the central metabolic drivers of androgen excess and reproductive dysfunction in PCOS [6]. Clinical studies have shown that exenatide, especially when combined with metformin, significantly lowers insulin resistance (Homeostatic Model Assessment of Insulin Resistance (HOMA-IR)) compared with metformin alone [6,13,14,15]. In a study assessing cardiovascular risk in women with PCOS, exenatide treatment significantly reduced C-reactive protein (CRP) levels (from 8.5 ± 1.4 to 5.6 ± 0.8 mmol/L, *P* = 0.001). This reduction in CRP correlated with improvements in insulin resistance, highlighting the combined metabolic and anti-inflammatory actions of exenatide that may further enhance reproductive outcomes [14].

Clinically, these combined hormonal, metabolic, and anti-inflammatory effects translate into more regular menstrual cycles, higher ovulation rates, and improved fertility outcomes [1,4,11]. Table 1 summarizes the effects of GLP-1 RA for IIH and PCOS.

**Table 1:** GLP-1 receptor agonists (GLP-1 RAs): mechanisms and outcomes in IIH and PCOS. This table summarises the mechanisms by which GLP-1 receptor agonists, particularly exenatide (the most studied), act in idiopathic intracranial hypertension (IIH) and polycystic ovary syndrome (PCOS). It integrates mechanistic, metabolic, hormonal, and clinical outcomes. Sources: [1-15]. GLP-1R, glucagon-like peptide-1 receptor; POMC, proopiomelanocortin; CART, cocaine- and amphetamine-regulated transcript; CSF, cerebrospinal fluid; ICP, intracranial pressure; NHE3, sodium-hydrogen exchanger 3; BP, blood pressure; SHBG, sex hormone-binding globulin; LH, luteinizing hormone; FSH, follicle-stimulating hormone; DHEAS, dehydroepiandrosterone sulfate; FAI, free androgen index; CRP, C-reactive protein; HOMA-IR, Homeostatic Model Assessment of Insulin Resistance; CVD, cardiovascular disease

Mechanism/site of action	Effect in IIH (with exenatide)	Effect in PCOS (with exenatide)	Key hormonal/clinical outcomes
Appetite suppression and delayed gastric emptying (activation of GLP-1 receptors in hypothalamic POMC/CART pathways) [1,12,13]	Weight loss leads to a reduction in intracranial pressure (ICP) [1]	Weight loss improves insulin resistance and lowers androgen levels [6]	Decreased BMI, decreased waist circumference, decreased visceral fat
Direct CSF secretion effects (GLP-1R in choroid plexus) [4,5]	Exenatide has been shown in rodent studies to reduce CSF secretion [4]. In human RCTs, it significantly lowered ICP in women with IIH and reduced headache frequency [5].	-	Reduced ICP beyond the weight loss effect
Renal natriuretic/diuretic effect (decreased NHE3 activity in proximal tubule) [4]	Promotes natriuresis and fluid loss, contributing to ICP reduction	Supports metabolic control and modest blood pressure/volume regulation	Improved sodium and fluid balance
Improved insulin sensitivity [6,11-15]	-	Decreased hyperinsulinemia reduces theca cell androgen synthesis; increased SHBG restores the LH/FSH ratio.	Decreased insulin, decreased HOMA-IR, increased SHBG, and improved ovulation.
Hormonal regulation [6,11-15]	-	Decreased total testosterone, decreased free testosterone, decreased androstenedione, decreased DHEAS; increased SHBG; decreased FAI; normalises LH/FSH.	Restored follicular development, menstrual regularity, and improved fertility
Anti-inflammatory effects (decreased CRP, cytokines) [14]	-	Decreased CRP and systemic inflammation improve insulin sensitivity and ovulation.	Improved metabolic and reproductive outcomes
Direct ovarian effects (GLP-1R in ovarian tissue) [13]	-	Regulates granulosa cell activity and enhances follicular maturation and ovulation	Improved menstrual cyclicity and fertility
Symptom improvement (clinical outcomes) [4,11,12]	Improved headache frequency and severity; better vision-related quality of life [4]	Meta-analyses show improved pregnancy rates compared with metformin (exenatide) [11,12]	Higher ovulation and pregnancy rates, improved quality of life
Metabolic profile improvements [14]	-	Decreased post-prandial glucose and triglycerides; improved cardiometabolic profile	Reduced long-term diabetes and cardiovascular disease risk

## Conclusions

IIH and PCOS are disorders that occur predominantly in obese women of reproductive age. Rapid weight gain is a major risk factor for IIH, which may present with headache, visual disturbance, and occasionally without papilledema. Weight loss remains the cornerstone of management in both conditions, and GLP-1 RAs offer an effective adjunct by inducing satiety and promoting sustained reductions in body weight. Exenatide, the most extensively studied GLP-1 RA, has demonstrated dual benefits. In IIH, it acts directly on GLP-1 receptors in the choroid plexus, reducing CSF secretion through Na⁺/K⁺-ATPase inhibition, thereby lowering ICP. In PCOS, exenatide improves insulin sensitivity, reduces compensatory hyperinsulinemia, and restores hypothalamic-pituitary-ovarian axis function. Importantly, GLP-1 receptors have also been identified in ovarian tissue, where exenatide may directly enhance granulosa cell activity, support follicular maturation, and promote ovulation. This leads to normalization of sex hormone balance, lowering of hyperandrogenism, improved ovulatory and menstrual regularity, and ultimately enhanced fertility outcomes. GLP-1 RAs are a promising therapeutic option warranting further study for their potential in rapid ICP reduction in IIH and their effects on ovarian function in PCOS. Overall, GLP-1 RAs emerge as a promising therapeutic option, warranting further study to investigate their direct effects on rapid ICP reduction in IIH and ovarian function in PCOS.
